# Comparison of predicted and measured axial length for ophthalmic lens design

**DOI:** 10.1371/journal.pone.0210387

**Published:** 2019-01-07

**Authors:** Hyeong-Su Kim, Dong-Sik Yu, Hyun Gug Cho, Byeong-Yeon Moon, Sang-Yeob Kim

**Affiliations:** 1 Department of Optometry, Kangwon National University, Samcheok, Korea; 2 Department of Optometry and Vision Science, Kyungwoon University, Gumi, Korea; University of Alabama at Birmingham, UNITED STATES

## Abstract

Ocular parameters have been applied to ophthalmic lens designs in order to satisfy individual wearers. An axial length (AL) of them can be used in individual ophthalmic lens designs. Our aim was to propose a reliable formula that predicts an individual’s AL using the corneal radius and refractive error, and to demonstrate the applicability of this formula. A total of 348 subjects underwent keratometry, objective and subjective refraction, and AL measurement. The formula of calculated AL for prediction obtained from the original Gullstrand simplified schematic eye: *calculated AL = (24*.*00 × aveK/7*.*80)—(SE × 0*.*40)*, where *aveK* and *SE* denote average corneal radius and spherical equivalent, respectively. Calculated AL was 24.50 ± 1.83 mm, which was 0.18 ± 0.47 mm longer than the measured value of 24.32 ± 1.73 mm (p < 0.001). The proportion showing the differences between the calculated and measured ALs were 284 eyes (40.8%) for 0.00–0.25 mm, 525 eyes (75.4%) for less than 0.50 mm, 665 eyes (95.5%) for less than 1.00 mm, and 31 eyes (4.5%) for more than 1.01 mm. Correlation coefficient showed a very high correlation between calculated and measured ALs (r = 0.967, p < 0.001), and higher in the myopic than in the hyperopic group. The mean difference was 0.18 mm; the 95% limit of agreement was +1.10—-0.75 mm in all groups. Agreement was better in hyperopic eyes than myopic eyes. Prediction from calculation of AL with a formula using the corneal radius and SE provides an alternative method to direct measurements of AL, especially in the restricted environment, which can’t use biometric equipment for personalized ophthalmic lens design.

## Introduction

**Fig 1:**
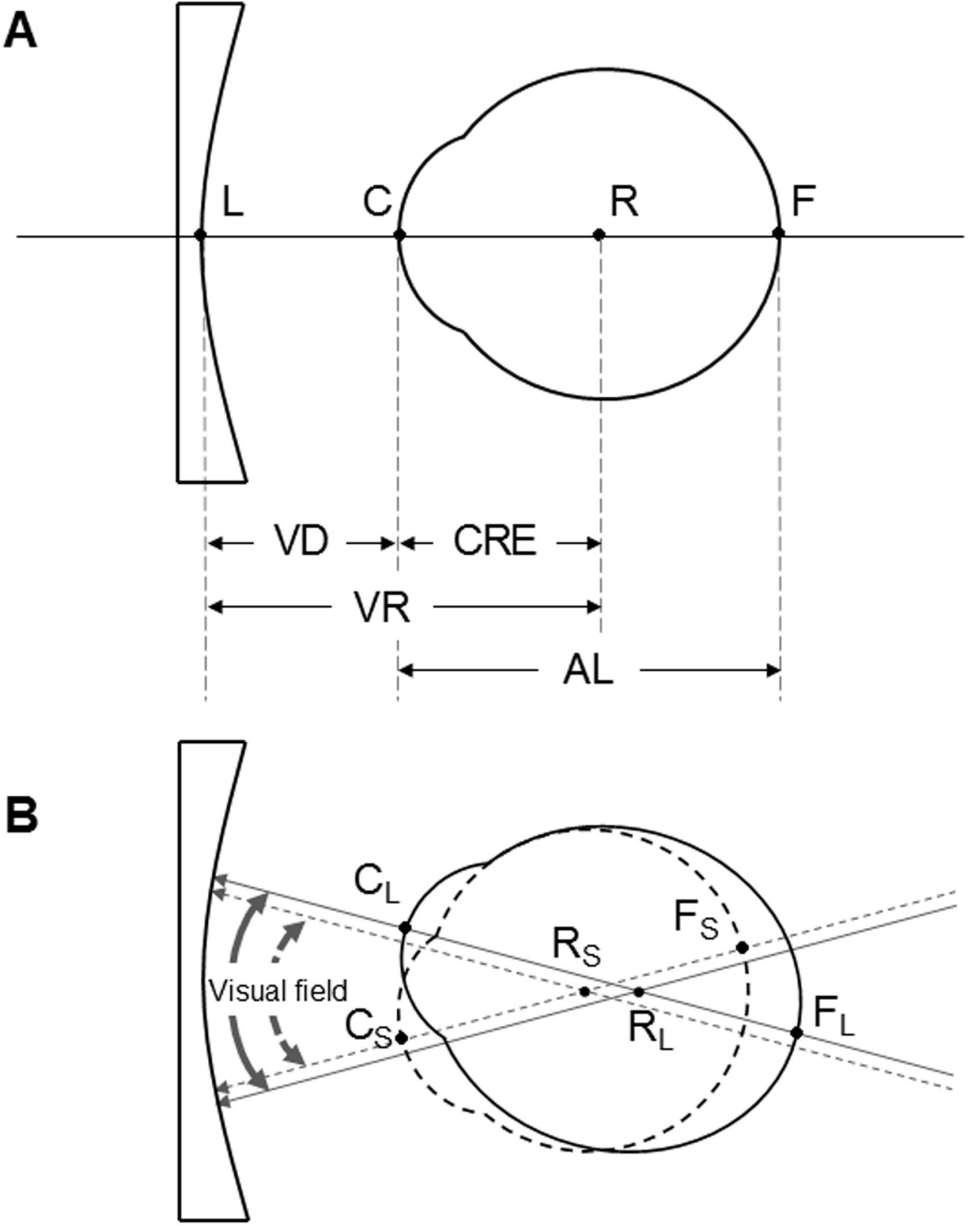
A schematic diagram of an optical model of an eye wearing spectacles (A) and the visual field of wearer with the axial length of the eye (B). VD is vertex distance; CRE is the distance from the vertex of the cornea (C) to the center of rotation of the eye (R); VR is the distance from the back vertex of the lens (L) to the center of the rotation of the eye; AL is the distance from the vertex of the cornea to the fovea of retina (F). Subscripts S and L mean small and large AL, respectively. Solid and dashed lines stand for large and small eye, respectively.

The eye is an ophthalmic optical system consisting of the cornea, crystalline lens, and vitreous body. When the components that enable the eye’s refractive power change, the refractive power of the whole eye is altered. In addition, reduction or increase of axial length (AL, defined as the length from the cornea to the fovea of retina, Fig 1A) is very important for determination of the refractive error of the eye [1]. The AL is approximately 16 mm at a newborn and reaches its full length of 22 to 25 mm at approximately 13 years of age [2]. At the age of 15, the anterior chamber depth reaches its longest length in consensus with the decreasing the refractive power of the crystalline lens, resulting in a decrease in the refractive power of the eye [3, 4]. An increase in AL and corresponding changes in the refractive power of the cornea and crystalline lens constitute the emmetropization mechanism [5–10]. Failure of the coordinated growth of these ocular components produces ametropia.

Progressive additional lenses (PALs) [11], along with bifocal and trifocal lenses, are used to correct near and distance visual acuity of presbyopia. Aves submitted the first patent application for the design of PALs in 1908 (cited by Meister *at al*. [12]). Maitenaz [13] devised a way to calculate each location of the lens and developed the first modern PALs known as Varilux (Essilor, France). Many studies have since been conducted, producing multiple types of PALs that allow a wider visual field and simultaneously minimize peripheral distortion. Production of personalized PALs began in the 21st century. The production of personalized PALs differs from previous methods by using a computer numerically controlled system to create the PAL surface. Therefore, it has become possible to produce PALs that can be optimized for each wearer. To make such a method feasible, lens manufacturers applied individual parameters of the eye, such as interpupillary distance, vertex distance (VD), pantoscopic angle, face form angle, and refraction results [14].

The personalization of ophthalmic lenses has been a market reality, and new parameters in ophthalmic lens design are needed to offer wearers increasingly lens efficiency and visual benefits [15]. The ‘Varilux Kan series’ of Physio New Edition, the progressive lens designed by Essilor, is made using different methods depending upon the AL that considers ametropia [16]. The AL is one of important parameters in designs for ophthalmic lens. Rotation of the eye relies on the position of the center of rotation CRE with the AL of the eye. Thus, an eye with large AL requires a wide visual field or an eye with small AL requires a narrow visual field when the rotation angle is the same (Fig 1B). Although the angle of rotation in myopic eye is small, the visual field through the ophthalmic lens is wide. In contrast to myopia, the visual field in hyperopic eye is narrow [16]. Therefore, ophthalmic lens need to be designed to achieve a higher performance for an individual wearer.

According to Yamakaji and Hatanaka [17], the base curve (BC) of ophthalmic lenses and the degree of insets (decentration of near optical center) can change depending on the VD and distance from the back vertex of the lens to the center of the VR. The degree of insets is important to the individualized design of ophthalmic lenses to minimize errors of refractive power and unnecessary prism effects. The degree of insets changes due to VR (it can be set according to AL) and BC. Each lens manufacturer strives to provide more comfort to wearers of both PALs and monofocal lenses. The measurements of the AL out of many individual parameters are not easy to carry out by ophthalmologists or optometrists under set-up of basic apparatus for refraction in primary eye care examination. In a restricted environment, it is necessary to contrive a method for predicting the AL using an autorefractor/keratometer and refractive error.

This study focused on the development of a formula that would incorporate easily measured factors, such as corneal radius and refractive error, to predict AL without the necessity of expensive methods such as applanation ultrasound and optical biometry [18], and to ascertain whether the formula for calculating AL was an applicable method for lens design of ametropic eyes.

## Materials and methods

### Subjects

There were 348 subjects (696 eyes, aged 46.17 ± 14.63 years) included in this study. Each subject provided informed consent to participate based on an oral explanation. This study was approved by Kangwon national university institutional review board (KWNUIRB-2017-12-003), and adhered to the tenets of the Declaration of Helsinki. Subjects were excluded due to previous ophthalmic surgery, history of trauma, presence of any ophthalmic condition that could alter AL measurements. Eyes were divided into three groups by spherical equivalent (SE) refractive error: the myopic group (SE ≤ -0.50 diopter (D)), hyperopic group (SE ≥ +0.50 D), and emmetropic group (-0.50 D < SE < +0.50 D).

### Procedure

All subjects underwent an optometric examination using an autorefractor/keratometer (HRK-7000, Huvitz Corp., Gunpo, Korea) for objective refraction and keratometry, subjective refraction for uncorrected visual acuity (UCVA) and corrected visual acuity (CVA), and the IOL-Master (IOL Master 500, version 7.5, Carl Zeiss Meditec Inc., Jena, Germany) for AL. For the main purpose of this study, a formula based on the Gullstrand simplified schematic eye was applied to the calculation of individual AL and was tested for applicability.

For the first derivation of the equation, a corneal radius of 7.8 mm and an AL of 24.0 mm were used in the original Gullstrand simplified schematic eye [19], and a proportional expression was developed. The compensation constant value of 0.4 (mm/diopter) for refractive error changed the AL substituted into the proportional expression, resulting in the final formula for calculated AL. According to Rubin, a change of 0.4 mm in AL equals a change of 1.00 D in refractive error in the case of axial ametropia [20, 21]. In addition, Lancaster [22] found that an increase of 1 mm in AL results in a change of 2.50 to 3.00 D. Nassaralla and Nassaralla [23] stated that a change of 1 mm in AL corresponds to a refractive change of 2.45 D. Therefore, we applied the compensation constant of 0.4 to the formula for calculated AL (calAL). AL and CR in normal eye of the Gullstrand simplified schematic eye are 24.00 mm and 7.8 mm, respectively. It is assumed that axial length is proportional to corneal radius of curvature. Unknown AL in an eye can be set as calAL, and measured CR in an eye can be set as aveK. Two proportional relationship can be set equal to each other. Hence, first term is 24.00 × aveK/7.80.

The formula for prediction is as follows:

calAL=(24.00×aveK/7.80)−(SE×0.40) (1)

where aveK is the average corneal radius and SE is spherical equivalent. For example, the AL of an individual with an aveK of 7.46 mm and an SE of -4.50 D (subjective refraction value) can be calculated using the given formula:

calAL=(24.00×7.46/7.80)−(−4.50×0.40)=24.75mm

The AL measurement obtained by the IOL-Master was 24.96 mm. Thus, the difference between the methods was 0.21 mm. The calALs from Eq (1) were compared to those measured with IOL-master.

### Data analysis

Data were collected and analysed using MedCalc software version 12.7.7.0 (MedCalc Software, Mariakerke, Belgium). The mean difference, distribution of difference, and Pearson correlation coefficient between calculated and measured AL were evaluated for each group. A paired t-test was used to compare the means between the two AL determination methods in each group. The linear regression of the measured ALs and refractive errors was performed to verify constant compensation, which was a rate of change in AL per diopter. Bland–Altman plots were used to evaluate the AL agreement between two the methods with 95% confidence intervals (CI). A p-value of ≤ 0.05 was considered significant.

## Results

The demographic data are shown in Table 1. A total of 696 eyes of 348 subjects were divided into 3 groups. The myopic group included 415 eyes (59.6%), the hyperopic group, 196 eyes (28.2%), and the emmetropic group, 85 eyes (12.2%). The SE of the myopic group was -4.44 ± 3.62 D (mean ± SD); the hyperopic group was +1.47 ± 1.36 D and the emmetropic group was -0.25 ± 0.24 D. Distribution of cylindrical power are 34 eyes (4.9%) for zero, 453 eyes (65.1%) for ≤ − 1.00 D. 158 eyes (22.7%) for ≤ − 2.00 D, 32 eyes (4.6%) for ≤ − 3.00 D, and 19 eyes (2.7%) for ≤ − 4.75 D.

**Table 1:** Demographic and refractive characteristics of subjects.

Characteristics	Values
**Number of subjects / eyes**	**348 / 696**
**Mean age (mean ± SD, year)**	**46.17 ± 14.63**
**Age range (year)**	**6 to 77**
**Gender (male / female)**	**158 / 190**
**Distance visual acuity (mean ± SD, decimal)**	****
** Uncorrected**	**0.37 ± 0.35**
** Corrected**	**1.01 ± 0.14**
**Refractive error (mean ± SD, D)**	****
** Sph**	**-1.75 ± 3.87**
** Cyl**	**-1.02 ± 0.79 (0 D to − 4.75 D)**
** SE**	**-2.24 ± 3.96**
**Distribution of refractive error**^a^ **(eyes)**	**Myopia / hyperopia (%)**
** ± 0.5 D ≤ SE < ± 2.5 D**	**133 (32.0%) / 172 (87.8%)**
** ± 2.5 D ≤ SE < ± 5.0 D**	**159 (38.3%) / 16 (8.1%)**
** ± 5.0 D ≤ SE < ± 10.0 D**	**97 (23.4%) / 8 (4.1%)**
** ± 10.0 D≤ SE**	**26 (6.3%) / 0 (0%)**

SD: standard deviation; Sph: spherical power; Cyl: cylindrical power; SE: spherical equivalent.

^**a**^Plus and minus sign indicate hyperopic and myopic eye, respectively.

The mean and SD of calculated and measured ALs of the three groups are presented in Table 2. There were no significant differences between calculated and measured AL in the hyperopic or emmetropic groups (paired t-test p = 0.080; p = 0.637, respectively). In the myopic group, the calculated AL was a statistically significant 0.27 mm longer than the measured AL (p < 0.001). Overall, the calculated AL was 0.18 mm longer than measured AL (p < 0.001).

**Table 2:** Differences between calculated and measured AL (n = 696 eyes).

	Calculated AL	Measured AL	Difference	p-value
**Myopia (mean ± SD, mm)**	**25.46 ± 1.66**	**25.19 ± 1.61**	0.27 ± 0.49*	**p < 0.001**
**Hyperopia (mean ± SD, mm)**	**22.94 ± 0.99**	**22.88 ± 0.95**	**0.05 ± 0.43**	**p = 0.080**
**Emmetropia (mean ± SD, mm)**	**23.43 ± 0.70**	**23.41 ± 0.69**	**0.02 ± 0.36**	**p = 0.637**
**Total (mean ± SD, mm)**	**24.50 ± 1.83**	**24.32 ± 1.73**	0.18 ± 0.47*	**p < 0.001**

*p < 0.05 by paired t-test was considered significant.

AL: axial length; SD: standard deviation.

Distributions of differences between the calculated and measured results are shown in Table 3. Of 696 eyes, 432 (62.1%) had a calculated AL longer than the measured AL; 264 (37.9%) eyes had a shorter calculated AL than measured AL. A difference of less than 0.5 mm was observed in 68.4% of the myopic group, 82.7% of the hyperopic group, and 92.9% of the emmetropic group. A difference of less than 1.0 mm was observed in 92.8% of the myopic group, 99.5% of the hyperopic group, and 100% of the emmetropic group. Overall, in 75.4% of eyes, there was a difference between the two methods of less than 0.5 mm; in 95.5% the difference was less than 1.0 mm; in 4.3% (30 eyes) the calculated AL was 1.01 mm or longer than the measured ALs, and in 1 eye (0.1%) the measured AL was 1.01 mm or shorter than the calculated AL.

**Table 3:** Distribution of differences between calculated and measured AL according to refractive error for each eye.

Differences (mm)	Myopia		Hyperopia		Emmetropia		All groups (%)		
	Over	Under	Over	Under	Over	Under	Over	Under	Total
**0.00–0.25**	**80**	**75**	**43**	**43**	**24**	**19**	**147 (34.0)**	**137 (51.9)**	**284 (40.8)**
**0.26–0.50**	**87**	**42**	**41**	**35**	**15**	**21**	**143 (33.1)**	**98 (37.1)**	**241 (34.6)**
**0.51–0.75**	**49**	**10**	**17**	**11**	**2**	**1**	**68 (15.7)**	**22 (8.3)**	**90 (12.9)**
**0.76–1.00**	**40**	**2**	**3**	**2**	**1**	**2**	**44 (10.2)**	**6 (2.27)**	**50 (7.2)**
****≥** 1.01**	**30**	**0**	**0**	**1**	**0**	**0**	**30 (6.9)**	**1 (0.4)**	**31 (4.5)**
**All**	**286**	**129**	**104**	**92**	**42**	**43**	**432 (62.1)**	**264 (37.9)**	**696 (100)**

Over: calculated AL is longer than measured AL; Under: calculated AL is shorter than measured AL.

**Fig 2:**
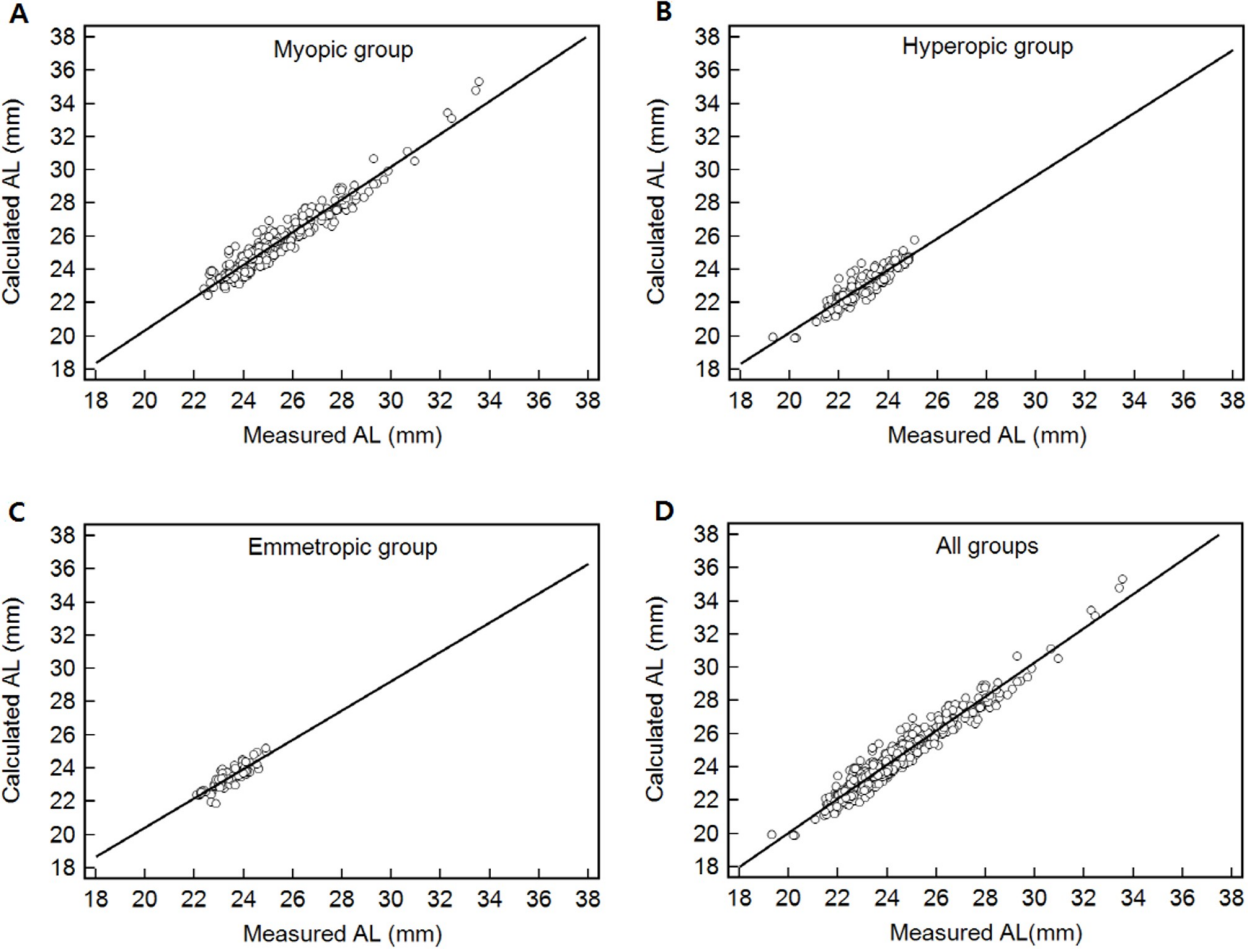
Correlation between calculated and measured axial length (AL) for each group. The lines in the scatterplots demonstrate the linear regression. (A) Myopic group (r = 0.955, p < 0.001). (B) Hyperopic group (r = 0.904, p < 0.001). (C) Emmetropic group (r = 0.871, p < 0.001). (D) All groups (r = 0.967, p < 0.001).

To elucidate the relationship between the calculated and measured ALs for each group, scatter diagrams and regression lines were plotted as shown in Fig 2. The statistically significant correlations between two methods were as follows: r = 0.955 for the myopic group, 0.904 for the hyperopic group, 0.871 for the emmetropic group, and 0.967 overall.

**Fig 3:**
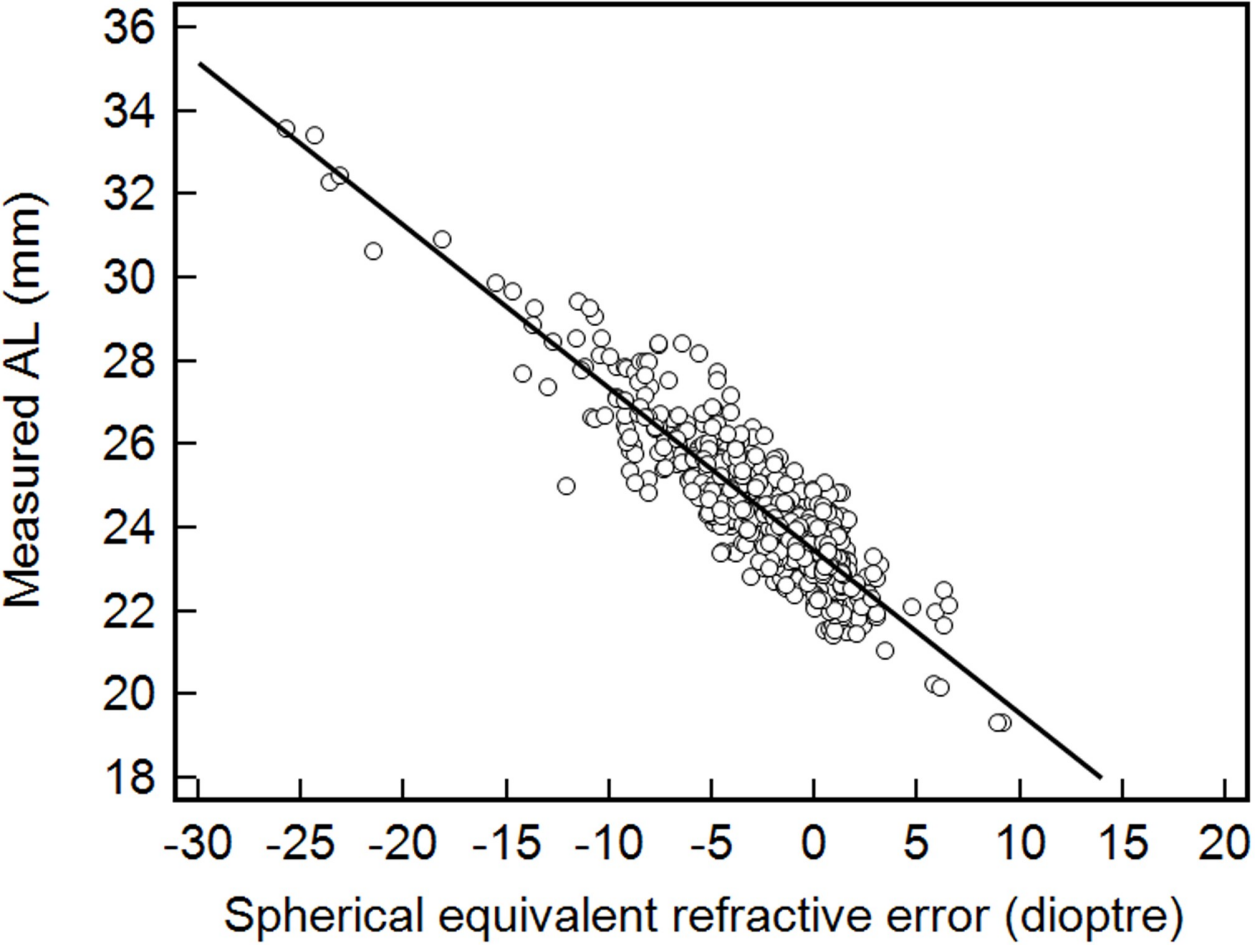
Linear regression plot of measured and spherical equivalent refractive error.

The scatter diagram and regression line of AL measurement and refractive error were constructed to identify the rate of change in AL with refractive error (Fig 3); and the obtained regression equation is shown (2):

AL=23.45−0.39×SE (2)

where AL = axial length and SE = spherical equivalent. Compensation constant was equal to 0.39.

**Fig 4:**
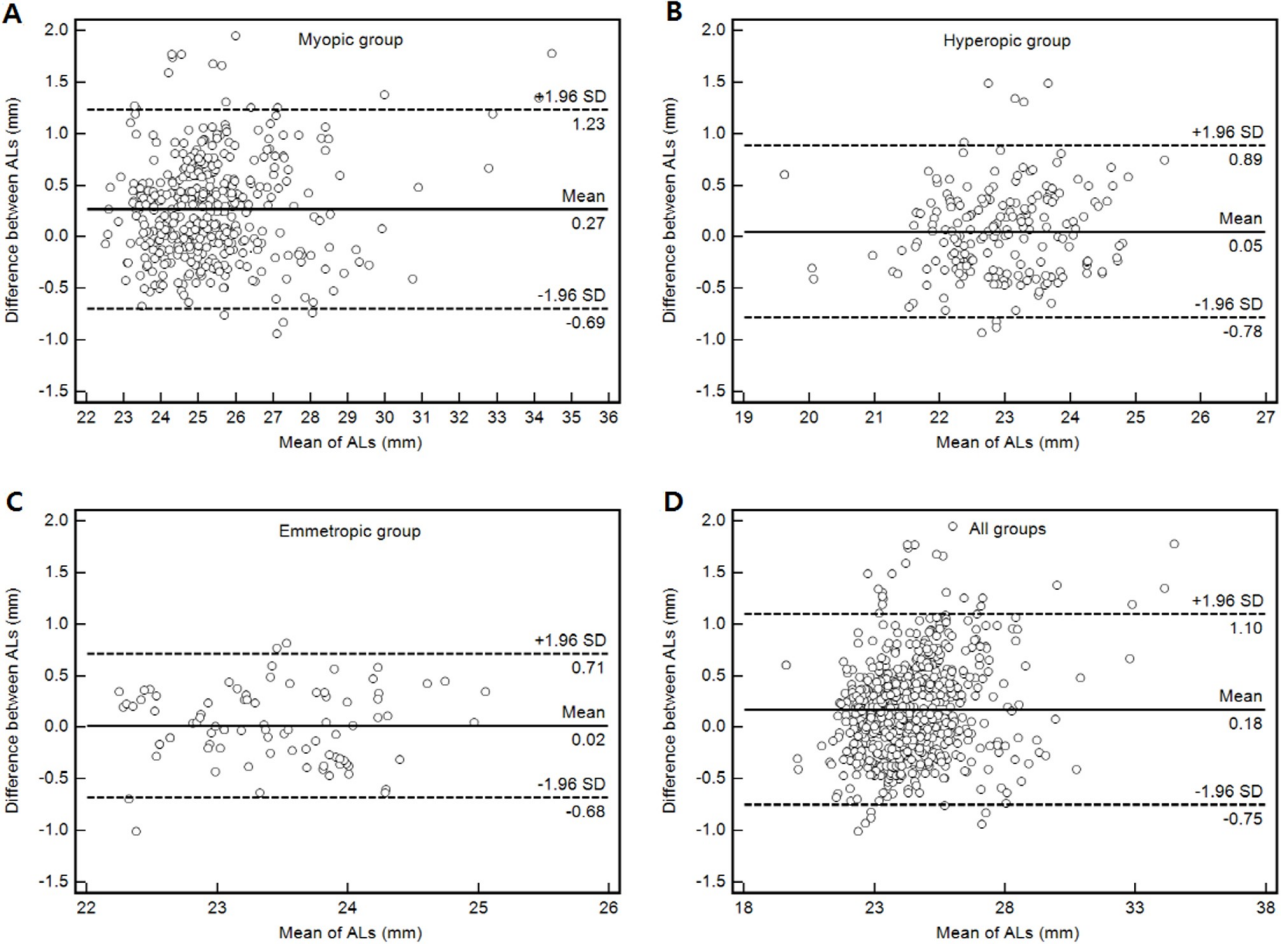
Plot of the mean differences between calculated and measured axial length against the mean of each. The dotted lines represent the upper and lower 95% limits of agreement (mean difference ± 1.96 × standard deviation (SD) of the differences). The solid line represents the mean of the differences. (A) Myopic group, (B) Hyperopic group, (C) Emmetropic group, (D) All groups.

Bland-Altman analyses were plotted as shown in Fig 4 to evaluate the agreement between the two methods. Similar results in the hyperopic and emmetropic groups were observed: mean differences were 0.05 mm and 0.02 mm, and 95% limits of agreement were +0.89 mm to -0.78 mm and +0.71 mm to -0.68 mm, respectively. Results for the myopic group and for the total of three groups were also similar: the mean differences were 0.27 mm and 0.18 mm, and the 95% limits of agreement were +1.23 mm to -0.69 mm and +1.10 mm to -0.75 mm, respectively.

## Discussion

AL is commonly used in the calculation of intraocular lens (IOL) power in patients undergoing cataract surgery, and to determine the extent of recess or resection in each extraocular muscle in patients undergoing strabismus surgery. Research regarding change of refractive power (myopic progression) also uses AL. AL measurement requires an applanation ultrasound and optical biometry, such as A-scan or IOL-Master. In environments lacking relatively expensive measurement apparatus, the optician or the optometrist have difficulty in obtaining the values of parameters, such as the individuals’ AL, at each primary eye care examination.; measurement of the AL each time lenses are prescribed is difficult and impractical under set-up of basic apparatus for refraction in primary eye care examination. In current practice, generally, only the refractive errors for emmetropia and ametropia are used for most off-the-shelf ophthalmic lens design. In addition, the patent documents mentioned earlier in this paper considered designing lenses according to the length of the CRE [16, 17], yet the only way to measure AL is to use measuring facilities or a regression analysis function. However, it is difficult to measure every individual’s AL, and the use of a regression analysis function is still questionable.

Our results show that, in cases of emmetropia and ametropia, the ALs calculated using the Eq (1) may be able to predict ALs for ophthalmic lens design without optical biometry. In another calculation of AL from spherical refraction, a considerable difference was found between the actual measurement of AL and the calculated AL in Berthezene *et al*. [16] when the regression analysis function (d = -23.58–0.299SR, where d: axial length, SR: spherical refraction) [24, 25] was applied. Difference between calculated and measured AL was a mean of 1.41 ± 2.84 mm in comparison to the results of our study (0.18 ± 0.47 mm). Grosvenor and Scott [26] applied a different regression analysis function (AL/CR = 2.9988–0.05446e, where AL: axial length, CR: corneal radius, e: refractive error) and found a mean difference of 0.38 ± 0.50 mm, twice more than the mean difference in our study. Thus, this study should be used to solve the problems of cost, utility, and practicality by applying the formula for calculated AL based on the Gullstrand simplified schematic eye, which is well-known and widely used.

The compensation constant value for refractive error of 0.4 in Eq (1) was determined based on previous studies [20–23]. In this regression analysis function, the change of refractive power in relation to AL was similar to previous studies. Thus, a change of 0.39 mm in AL is equal to a change of 1.00 D in refractive error as Eq (2). Therefore, it was reasonable to use 0.4 for the refractive error compensation constant. Kushner *et al*. [24] created a formula to calculate AL when the A-scan was unable to determine how far to recess or resect each individual extraocular muscle in strabismus surgery. The formula for calculated AL was developed using a linear regression function of refractive error and age. In Kushner's study, a difference in AL of less than 0.5 mm was observed in 43% of total participants, 75% had a difference of less than 1.0 mm, and 92% had a difference of less than 1.5 mm. In this study, 75.4% of total participants had a difference in AL of less than 0.5 mm, and 95.5% had a difference of less than 1.0 mm. Results demonstrated a higher rate of accuracy compared to than those in Kushner’s experiment. The reasons for such accuracy were: first, Kushner’s research used a simple regression analysis function based on the participants of the study and second, his function only included refractive error and age, not corneal radius.

The accuracy of calculated ALs to measured ALs is variable according to groups of refractive errors, but in all groups (Figs 2D and 4D), the regress equation y (calculated AL) = -0.432 + 1.025x (measured AL) (r = 0.967, p < 0.001) was verified with 696 eyes. In addition, the coefficient of determination (R^2^) was 93.4%, which suggests the controllable variables were high. In the Bland-Altman analyses, a range of +1.10 mm was determined as the maximal difference that could be agreed upon between calculated and measured methods. The calculated value was longer than the actual measured value. The SD for difference between both methods was less than 0.47 mm.

The limitation of this study is that the exact difference or range of difference between the calculated and measured AL as a useful factor of lens design is not specified because the tolerance limit of AL in lens design based on AL remains unknown. However, Bleshøy’s study showed that lens design may give an indication on the effect of using individual designs for persons who deviate more than ± 1 mm from the standard CRE [27]. If the standard CRE is replaced with the AL, the deviation of the AL is equal to ± 1.85 mm because the ratio of AL to CRE in normal eye is 24 mm to 13mm. Therefore, the ± 1 mm difference between the calculated and measured AL is acceptable errors. The majority of calculated ALs remains within tolerable limits. Berthezene *et al*. [16] also found that the field width could be increased by 5% when the AL of the eye was elongated by 10% (ca. 2.4 mm of 24 mm). The outcome of the current study consists of 95.5% within 1.0 mm (4.2% of 24 mm) of the calculated AL. Hence, the variation of the field width may have been small, within 2.1%. The proposed equation may be also some limitation for application to eye with cataract or lenticular astigmatism. Another limitation was that age was not considered as a factor. In general, AL increases rapidly early in life, then more slowly until adulthood, and decreases in old age [28]. Thus, older participants are likely to have shorter ALs than younger participants [29]. However, people with myopia tend to have a longer axial length, and people with hyperopia tend to have a shorter axial length compared to those with emmetropia. For this reason, most agree that AL is the largest determinant of refractive error [30]. In our results, which were determined using the corneal radius and SE, the correlation of calculated AL using measured AL was higher in ametropic groups than in the emmetropic group.

Unlike past studies, many individual parameters are considered in ophthalmic lens design, as is AL. Endeavors to apply AL to ophthalmic lens design are still going on how to increase the comfort of wearing spectacles. AL can be measured using expensive biometric systems such as A-scan or IOL-master. Yet, opticians or optometrists must face the cost of acquiring such machines and the difficulty of measuring each individual’s AL. Our study has shown that this formula for predicting the individuals’ AL, without the need for direct methods such as applanation ultrasound or optical biometry, provides an economic alternative to design customized ophthalmic lenses for individuals with ametropic eyes. Furthermore, by calculating AL, it will become possible for lens manufacturers to design and produce more precise ophthalmic lenses using the formula, and ophthalmologists or optometrists will be able to help wearers use ophthalmic lenses more comfortably.

## Supporting information

S1 File All relevant raw data. (XLSX)
